# Cerebral malperfusion resolution after repair of acute DeBakey type I dissection with a novel hybrid prosthesis: early results of the PERSEVERE Study^^†^^

**DOI:** 10.1093/ejcts/ezaf199

**Published:** 2025-06-30

**Authors:** William Brinkman, John J Squiers, Arminder Jassar, Shinichi Fukuhara, Fernando Fleischman, Hiroo Takayama, Ibrahim Sultan, George Arnaoutakis, Michael C Moon, Wilson Y Szeto, PERSEVERE Investigators, Joshua Grimm, John Frederick, Patrick Vargo, Brad Leshnower, Mohiuddin Cheema, Basel Ramlawi, Sanford Zeigler, Joseph DeRose, Ismail El-Hamamsy, Derek Brinster, Chris Malaisrie, Castigliano Bhamidipati, Claire Watkins, Kyle Eudailey, T Brett Reece, Eric Jeng, Puja Kachroo, Prashanth Vallabhajosyula

**Affiliations:** Department of Cardiothoracic Surgery, Baylor Scott & White The Heart Hospital Plano, Plano, TX, USA; Department of Cardiothoracic Surgery, Baylor Scott & White The Heart Hospital Plano, Plano, TX, USA; Cardiac Surgery, Massachusetts General Hospital, Boston, MA, USA; Department of Cardiac Surgery, University of Michigan, Ann Arbor, MI, USA; USC Cardiac and Vascular Institute, University of Southern California, Los Angeles, CA, USA; Division of Cardiac, Thoracic & Vascular Surgery, Columbia University, New York, NY, USA; Division of Cardiac Surgery, University of Pittsburgh School of Medicine, University of Pittsburgh Medical Center, Pittsburgh, PA, USA; Division of Cardiovascular and Thoracic Surgery, Dell Medical School, University of Texas at Austin, Austin, TX, USA; Division of Cardiac Surgery, Department of Surgery, University of Alberta, Edmonton, AB, Canada; Division of Cardiovascular Surgery, Perelman School of Medicine, University of Pennsylvania, Philadelphia, PA, USA

**Keywords:** Acute DeBakey type I aortic dissection, Hemiarch repair, Cerebral malperfusion, Hybrid aortic prosthesis, False lumen thrombosis

## Abstract

**OBJECTIVES:**

Patients undergoing hemiarch repair for acute DeBakey type I dissection (ADTI) are high risk for postoperative stroke, especially if cerebral malperfusion is present preoperatively. We sought to evaluate whether the AMDS Hybrid Prosthesis (AMDS), a bare metal stent designed to promote positive aortic remodelling and prevent distal anastomotic new entry tears, may improve neurological outcomes of patients with ADTI presenting with cerebral malperfusion.

**METHODS:**

PERSEVERE enrolled patients presenting with ADTI and malperfusion at 26 sites in North America. Among 93 enrolled patients, 30 (32.3%) presented with cerebral malperfusion. We evaluated for resolution of clinical and/or radiological cerebral malperfusion after hemiarch repair with AMDS.

**RESULTS:**

Cerebral malperfusion was diagnosed clinically in 19 (63.3%) patients and radiographically in 23 (76.7%) patients. Among the patients with clinical cerebral malperfusion, 18 survived the perioperative period; of these, 11 (61%) experienced complete resolution of preoperative symptoms, 5 (28%) had no worsening of preoperative symptoms, and 2 (11%) had a new disabling stroke postoperatively. At follow-up, the mean true lumen to total arterial diameter ratio (measured by computed tomography angiography) improved from 30.9% to 64.4% (*P* = 0.002) in the innominate artery and 33.8% to 60.6% (*P* = 0.005) in the left common carotid artery from preoperative baseline in patients with radiographic cerebral malperfusion.

**CONCLUSIONS:**

Among patients presenting with ADTI and cerebral malperfusion, the majority had resolution or stability of neurological symptoms after hemiarch repair using the AMDS. Radiographic indicators of malperfusion also improved.

**CLINICAL TRIAL REGISTRATION:**

https://clinicaltrials.gov/study/NCT05174767

## Graphical Abstract

**Figure:**
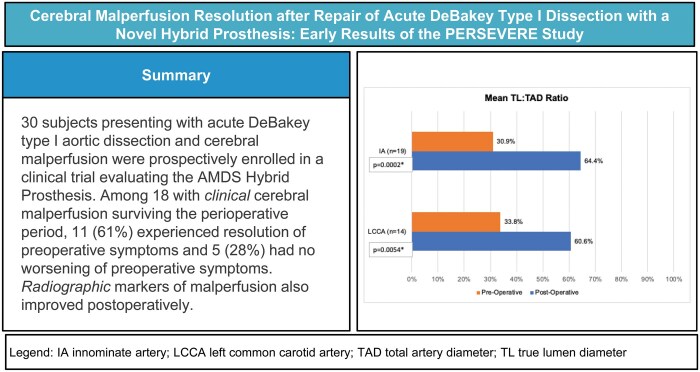

## INTRODUCTION

Among patients with acute DeBakey type I dissection (ADTI), those presenting with preoperative malperfusion have increased risks of morbidity and mortality [1]. Previous registry reports have shown that between 5% and 15% of patients undergoing surgery for type A aortic dissection present with cerebral malperfusion, with postoperative stroke rates in these patients ranging from 17% to 23% [2–5]. However, reasonable long-term survival can be achieved for patients with preoperative cerebral malperfusion undergoing repair of ADTI [6, 7].

The AMDS Hybrid Prosthesis (Artivion, Atlanta, GA, USA) is a self-expanding, open-cell Nitinol stent with a proximal polytetrafluoroethylene (PTFE) cuff deployed antegrade into the arch and descending aorta during circulatory arrest as an adjunct to proximal aortic reconstruction in patients with ADTI. This prosthesis is designed to stabilize the true lumen (TL), avoid distal anastomotic new entry tears (DANE) and enhance aortic arch remodelling. The PERSEVERE trial enrolled subjects with ADTI and preoperative malperfusion undergoing proximal aortic repair with deployment of the AMDS device. Early results from PERSEVERE have demonstrated reduced rates of major adverse events (MAEs) and DANE at 30 days compared to reference rates from registry studies [8].

Herein we report a sub-study of PERSEVERE subjects who specifically presented with cerebral malperfusion. We aimed to evaluate for resolution of clinical and/or radiographic signs of cerebral malperfusion after proximal aortic repair and deployment of the AMDS device.

## PATIENTS AND METHODS

### Study design and end-points

The PERSEVERE study (NCT05174767) is a prospective, single-arm, nonblinded, investigation trial conducted at 26 centres in North America that enrolled 93 adult patients presenting with ADTI and undergoing proximal aortic repair with deployment of the AMDS Hybrid Prosthesis. Details of study design, including inclusion/exclusion criteria, and the early primary results of the trail have recently been published elsewhere [8]. Of note, patients were eligible for enrollment only if the primary entry tear was located in the ascending aorta [at least 1.5 cm proximal to the innominate artery (IA) origin]; patients with significant reentry tears in the arch or proximal descending thoracic aorta were not eligible for enrollment.

Each study site received approval to enroll patients from the overseeing Institutional Review Board (central IRB reference number 20216348; initial approval on 17 December 2021). All subjects provided written informed consent and were enrolled between July 2022 and November 2023.

In agreement with the Food and Drug Administration (FDA) of the USA as part of an Investigational Device Exemption (IDE) submission, a reference cohort for the trial was established from 5 prior publications that reported individual MAE rates in patients with ADTI and clinical malperfusion syndromes [2, 9–12]. These publications were selected based on a systematic literature search for studies evaluating acute DeBakey Type I dissection in patients with preoperative malperfusion who were surgically treated with hemiarch repair and had data reported on these MAEs through 30 days or at least hospital discharge. A composite rate of MAE was estimated from these publications, and the average was considered the performance goal and primary end-point for the trial. Among these publications, the average rate of new postoperative stroke was 20.9%.

This study is an unspecified exploratory analysis of the PERSEVERE study. Although we did not intend for this analysis to impact conduct of the prespecified PERSEVERE analyses, we recognized the conduct and results of this study may have unforeseeable consequences on the prespecified analyses. Nevertheless, we elected to proceed with this analysis because we believed the potential benefits of an opportunity to report on prospectively-collected, detailed radiographic data on a unique subcohort of the PERSEVERE trial outweighed any potential risks to the prespecified analyses.

Among enrolled subjects presenting with cerebral malperfusion, we sought to evaluate for resolution of clinical and/or radiographic cerebral malperfusion. Clinical and radiographic malperfusion were diagnosed at the discretion of treating investigators at local sites. There is no consensus definition in the literature for resolution of malperfusion in patients with ADTI. We defined resolution of clinical cerebral malperfusion as resolution or stability of clinical symptoms leading to a preoperative diagnosis of cerebral malperfusion at 30-day follow-up. We defined improvement of radiographic malperfusion as TL expansion, specifically documented by change in TL to total arterial diameter (TAD) ratio.

### Operative technique

Details of AMDS sizing and deployment have also been described previously [8]. Briefly, the device is primarily sized based on TAD in zone 1 [between the IA and left common carotid artery (LCCA)] with a second TAD measured at the level of the pulmonary artery bifurcation to select a straight or tapered configuration of the prosthesis. During circulatory arrest, implantation was performed during resection of the ascending aorta. At least 1 cm of native aortic tissue proximal to the IA is left intact to accommodate the cuff of the AMDS device, which is introduced into the TL of the arch and descending aorta in an antegrade fashion. After stent deployment, the delivery system is removed and the anastomotic cuff is secured to the proximal rim of native aorta with a running circumferential suture. A Dacron graft was then used for ascending aorta replacement and sewn to the cuff of the AMDS device with a second running circumferential suture.

### Study data and core laboratory imaging analysis

Baseline cerebral malperfusion details were reported by the investigator as clinical, radiographic or both based on patient’s clinical presentation and preoperative computed tomography angiography (CTA) scans. CTAs were collected and analysed by an independent Imaging Core Lab (Cleveland Clinic's Vascular Core Laboratory) using TeraRecon analysis software (TeraRecon, Durham, NC). The aortic zones were based on the Society for Vascular Surgery (SVS) and Society of Thoracic Surgeons (STS) reporting standards for type B aortic dissections [13, 14].

We developed several measures to assess for resolution of radiographic cerebral malperfusion. Aortic measurements, including TAD, TL diameter and false lumen (FL) diameter, were measured at the narrowest TL diameter within 2 cm from the ostium in the IA and LCCA. Change in TL: TAD ratio in the vessels of interest (IA and LCCA) was calculated between the postoperative measurements and the preoperative measurements. There was no surgeon-reported evidence of left subclavian artery malperfusion preoperatively, so this vessel was not assessed as part of this analysis. The right common carotid artery was not assessed due to limited imaging visibility in the neck, as the study protocol only required CTA of the chest/abdomen/pelvis for enrollment and most patients did not have preoperative CTA neck available for analysis.

Postoperative CTAs were collected between predischarge and 6 months. We preferentially analysed postoperative CTAs from the 30-day postoperative visit for this study. For 10 patients who did not obtain a CTA at this visit or did not complete this visit, we used predischarge or 6-month CTAs in lieu of the 30-day study. The median number of days from index procedure to follow-up CTA analysed in this study was 39 days (range 0–186 days), and all patients who survived the perioperative period completed follow-up during the specified study period.

FL thrombosis status was also assessed in the vessels of interest as completely thrombosed, partially thrombosed or patent; in some cases, completely thrombosed included no presence of FL post-procedure.

Clinical data were prospectively collected, and the radiographic datapoints used in this sub-study were retrospectively measured and analysed.

### Statistical analysis

Analyses used all available data at time of data export; data collection per the study protocol is ongoing, though this study represented an exploratory interim analysis not prespecified by the protocol. The summaries were included for all available data in patients with preoperative cerebral malperfusion. To compare pre- and postoperative values, a paired t-test was used. Although the sample size was small, a test for normality proved the data to be in the range of normality to perform parametric tests. Continuous variables were summarized using the number of observations (N), mean, standard deviation (SD), median, minimum (min) and maximum (max). For discrete variables, frequency count and percentages of patients were computed; a *P* value <0.05 was considered to be statistically significant.

Analyses and summary outputs were generated using SAS^®^ version 9.4. Supplemental analyses were performed using Microsoft^®^ Excel^®^ version 2108.

## RESULTS

**Figure 1:**
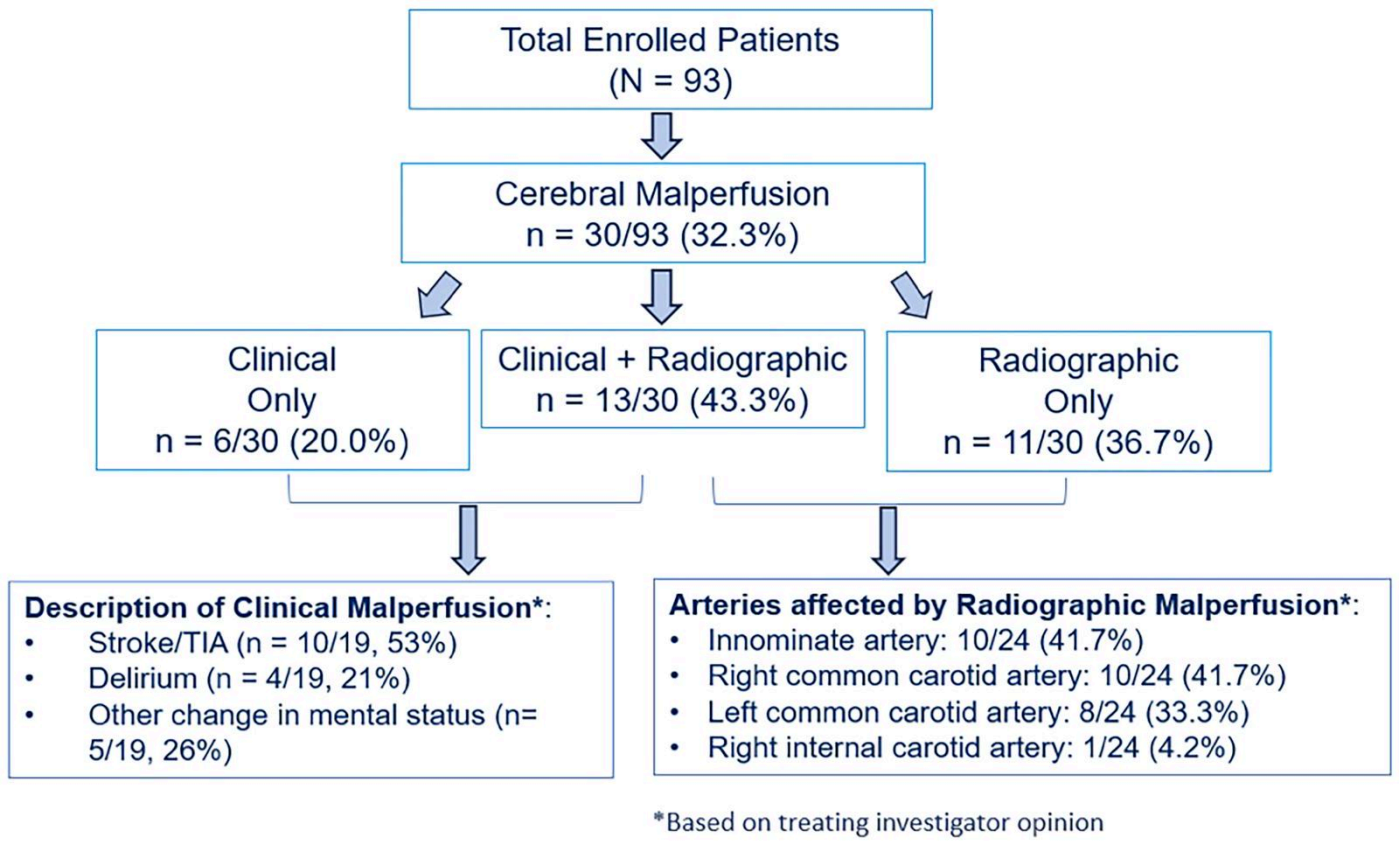
Patient selection flow diagram. Subjects presenting with clinical or radiographic (or both) signs of cerebral malperfusion were included in this sub-study of the PERSEVERE trial.

Among the 93 patients enrolled in the trial, 30 (32.3%) presented with either clinical or radiographic (or both) cerebral malperfusion. Cerebral malperfusion was diagnosed clinically in 19 (63.3%) patients and radiographically in 23 (76.7%) patients; 13 (43.3%) patients presented with both clinical and radiographic cerebral malperfusion (Fig. 1). Baseline characteristics of these patients are presented in Table 1.

**Table 1:** Baseline patient characteristics of patients enrolled in the PERSEVERE trial who presented with preoperative cerebral malperfusion

Characteristic	Value (*N* = 30 subjects)
Age at consent	
Median (min, max), years	60 (36, 78)
BMI	
Median (min, max), kg/m^2^	29.3 (18.0, 49.1)
Sex *n* (%)	
Males	22 (73.3%)
Females	8 (26.7%)
Race *n* (%)	
Asian	1 (3.3%)
Black or African American	6 (20.0%)
White	20 (66.7%)
Not reported	3 (10.0%)
Ethnicity *n* (%)	
Hispanic	1 (3.3%)
Non-Hispanic	26 (86.7%)
Not reported	3 (10.0%)
Medical history *n* (%)	
Arterial hypertension	11 (36.7%)
Hyperlipidemia	9 (30.0%)
History of chronic kidney disease	12 (40.0%)
Type II diabetes	4 (13.3%)
Peripheral arterial disease	0 (0.0%)
Cancer	6 (20.0%)
History of stroke or TIA	3 (10.0%)
Social history *n* (%)	
History of tobacco use	16 (53.3%)
History of illicit drug use	5 (16.7%)
Cerebral malperfusion *n* (%)	
Clinical only	6 (20.0%)
Radiographic only	11 (36.7%)
Clinical and radiographic	13 (43.3%)
Clinical malperfusion symptoms *n* (%) (*n* = 19)	
Stroke or TIA	10 (52.6%)
Delirium	4 (21.1%)
Other change in mental status	5 (26.3%)

BMI: body mass index; Hx: history; Max: maximum; Min: minimum; TIA: transient ischaemic attack.

Among the patients with cerebral malperfusion, median age was 60 years (range 36–78 years), and 22 (73.3%) were male. Patients with clinical cerebral malperfusion presented with the following symptoms: 52.6% new neurologic deficit of stroke or transient ischaemic attack (TIA), 21.1% delirium or 26.3% other changes in mental status such as altered mental status or dizziness. Operative characteristics of patients presenting with cerebral malperfusion are presented in Table 2. One patient with preoperative clinical cerebral malperfusion died prior to postoperative neurologic evaluation and was excluded from the sub-analysis.

**Table 2:** Operative characteristics of patients enrolled in the PERSEVERE trial who presented with preoperative cerebral malperfusion

Characteristic	Value (*N* = 30 subjects)
AMDS deployment time (min)	
Mean ± SD, median	4.6 (±2.97), 4.0
AMDS total implant time (min)	
Mean ± SD, median	15.3 (±5.86), 15.0
Hypothermic circulatory arrest time (min)	
Mean ± SD, median	30.9 (±20.29), 29.5
Cardiopulmonary bypass time (min)	
Mean ± SD, median	200.3 (±67.69), 187.5
Type of cerebral perfusion *n* (%)	
Antegrade	14 (46.7%)
Retrograde	15 (50.0%)
Unknown	1 (3.3%)
Concomitant procedures *n* (%)	
Yes	25 (83.3%)
Aortic root intervention	12 (40.0%)
Aortic valve resuspension	9 (30.0%)
Valve intervention	7 (23.3%)
Coronary artery bypass surgery	0 (0.0%)
Other	10 (33.3%)
Guidewire use *n* (%)	2 (6.7%)
Fluoroscopy use *n* (%)	1 (3.3%)
Method to identify the true lumen *n* (%)	
Transesophageal echocardiography	17 (56.7%)
Intravascular ultrasound	0 (0.0%)
Visual inspection	21 (70.0%)

AMDS: AMDS Hybrid Prosthesis; SD: standard deviation.

Resolution of clinical cerebral malperfusion was evident in 16/18 (89%) patients; 11 (69%) had complete resolution of preoperative symptoms leading to diagnosis of cerebral malperfusion, and 5 (28%) had no worsening of these preoperative symptoms. A new disabling stroke and/or worsening of preoperative symptoms leading to a diagnosis of cerebral malperfusion was diagnosed in 2/18 (11%) patients with preoperative cerebral malperfusion. Of note, among all enrolled patients, 9/93 (9.7%) patients had evidence of a new disabling stroke within 30 days, post-procedure.

**Figure 2:**
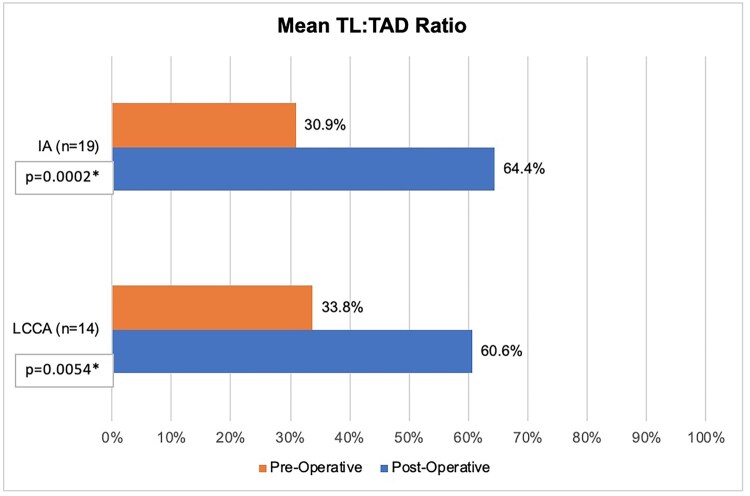
Mean true lumen to total arterial diameter (TL: TAD) ratio, expressed by percentage, as measured on computed tomography angiography at preoperative baseline and 30 days postoperatively in patients with acute DeBakey type I aortic dissection and radiographic cerebral malperfusion evident in the innominate artery (IA) or left common carotid artery (LCCA).

**Figure 3:**
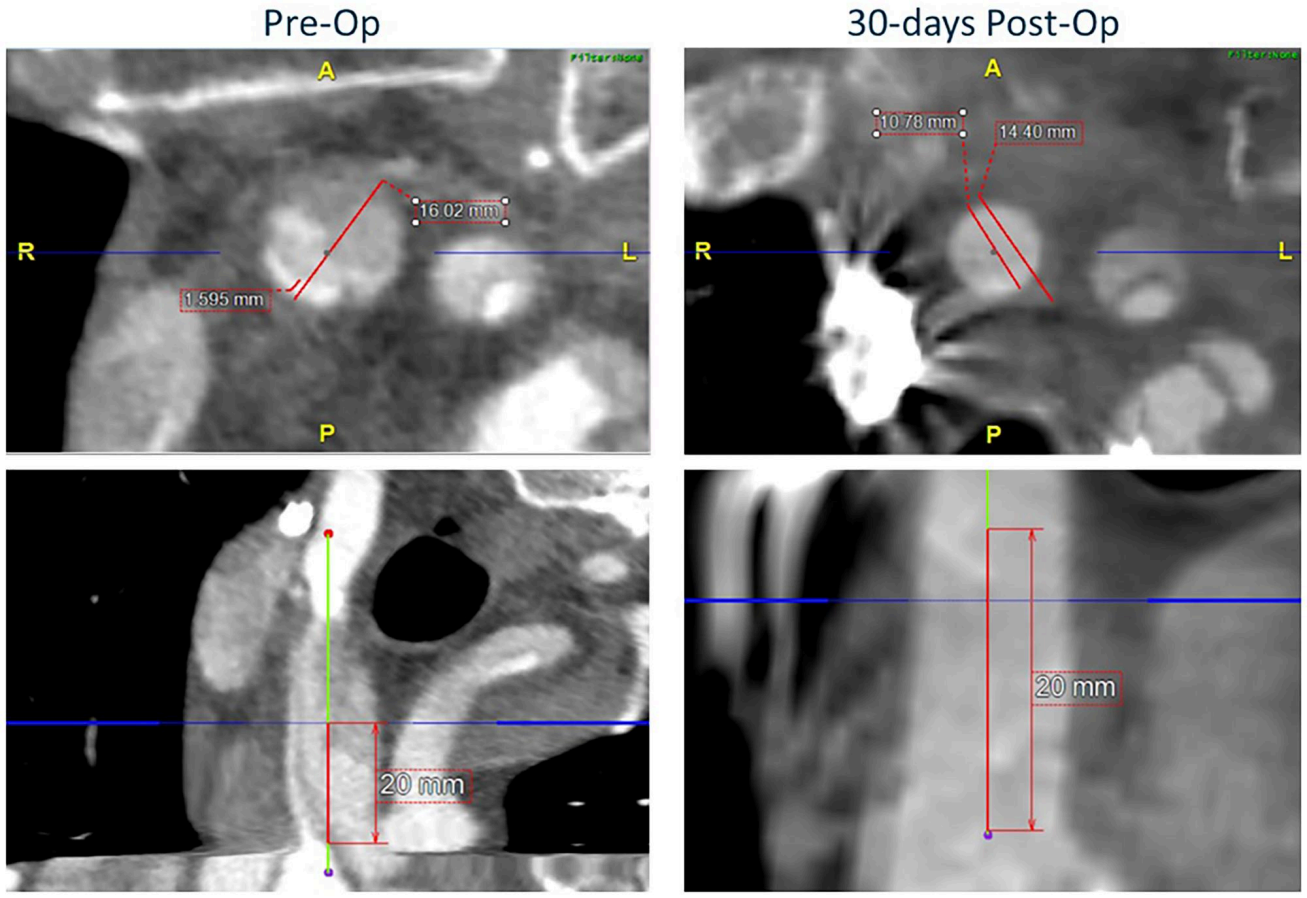
Representative core lab imaging measurements demonstrating improvement in true lumen to total arterial diameter ratio in the innominate artery from preoperative baseline to the 30-day postoperative timepoint.

**Figure 4:**
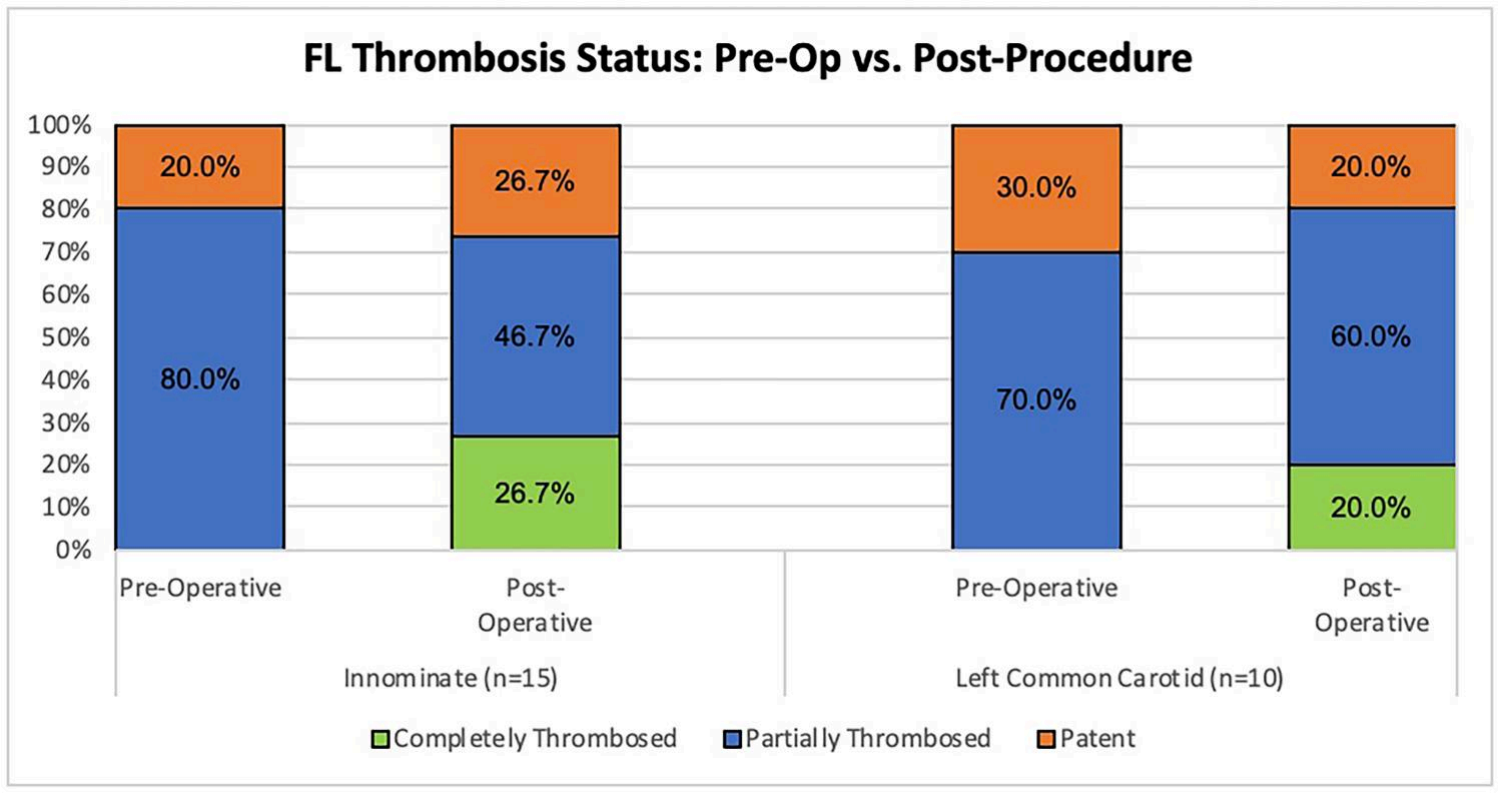
Rates of false lumen thrombosis and patency in the innominate and left common carotid arteries of patients presenting with acute DeBakey type I aortic dissection and radiographic cerebral malperfusion from preoperative baseline to 30-day postoperative timepoint. Thrombosis was assessed on computed tomography angiography by an independent imaging core laboratory.

Based on Core Lab review and among 23 patients with radiographic cerebral malperfusion, malperfusion was evident in 19 IA and 14 LCCA. The mean TL: TAD ratio from the preoperative CTA to postoperative CTA improved in both arteries (Fig. 2). Mean TL: TAD ratio improved from 30.9% to 64.4% in the IA (*P* = 0.002; range −30 – 87.8%; 95% confidence interval of a percentage change 18.0–48.9%) and from 33.8% to 60.6% in the LCCA (*P* = 0.005; range −6.6 – 100%; 95% confidence interval of a percentage change 9.4–44.1%). A representative example of improvement in TL: TAD ratio of the IA from baseline to the 30-day postoperative timepoint is presented in Fig. 3. Improvements in FL thrombosis were also generally noted in both the IA and LCCA, though 2 patients were graded as having newly patent FL channels postoperatively (Fig. 4).

## DISCUSSION

The PERSEVERE trial enrolled subjects presenting with ADTI and preoperative malperfusion undergoing proximal aortic repair with deployment of the AMDS device [8]. In this unspecified exploratory interim analysis, we have reported the preoperative characteristics and postoperative outcomes in the subset of patients enrolled in this trial who presented specifically with cerebral malperfusion (clinical, radiographic or both). Our main findings were 2-fold. First, clinical outcomes in regards to resolution of cerebral malperfusion and avoidance of new postoperative stroke were promising and represented an improvement compared to historical control data. Second, systematic evaluation of pre- and postoperative cross-sectional imaging demonstrated improvement in radiographic measures of cerebral malperfusion.

A limitation of the trial, and thus this analysis, is that no control of subjects was enrolled for comparison. However, there are ample available data regarding outcomes of patients presenting with ADTI and cerebral malperfusion, including from case series selected by the FDA as a reference cohort in their evaluation of the AMDS device from which the expected rate of new postoperative stroke was estimated at 20.9% [2, 9–12]. Additional recent reports may be informative as well. For example, a recent report from the STS Adult Cardiac Surgery Database demonstrated mortality rates approaching 30% among patients undergoing repair of a type A aortic dissection who presented with cerebral malperfusion, though postoperative stroke rates in this group were not specified [15]. Also, the latest data from the International Registry of Aortic Dissection (IRAD), which was published after FDA approval of the PERSEVERE trial, reported a postoperative rate of new cerebrovascular accident of 17.5% [4]. This IRAD cohort included approximately 20% of patients undergoing total arch replacement, which limits comparisons to the current study, in which patients underwent hemiarch repairs; nevertheless, we posit the postoperative rate of new stroke of 11% in the current study is promising in the context of the FDA-approved reference cohort outcomes.

It is also interesting to note that the new postoperative stroke rate in our study cohort was nearly identical to the rate of new postoperative stroke among the other patients [without preoperative cerebral malperfusion but with other malperfusion syndrome(s)] enrolled in the PERSEVERE trial. The similar rate of new postoperative stroke among both cohorts (those *with* and *without* preoperative cerebral malperfusion) suggests that postoperative strokes diagnosed in our study cohort may be more likely due to risks inherent to repair of type A dissection, including embolic phenomena and preoperative static vessel obstruction, rather than continued dynamic cerebral malperfusion. We feel the data are hypothesis-generating evidence pointing to the benefit of AMDS in the treatment of dynamic cerebral malperfusion.

Although, due to the study design lacking a control cohort, we cannot definitively conclude the ADTI repair using the AMDS device as an adjunct to hemiarch replacement reduces postoperative stroke rates compared to hemiarch replacement alone [16], it is our opinion that the combination of enhanced immediate expansion of the TL, and a reduction in DANE may play a role in preventing post-repair persistent cerebral malperfusion that leads to postoperative stroke. In the primary PERSEVERE trial, no DANE was observed within 30 days of surgery, which was a substantial reduction compared to the expected rate of 45% based on the reference cohort [8]. Prevention of DANE may also encourage positive aortic remodelling, which in this sub-study may have led to the improvements in the radiographic end-points selected to assess for cerebral malperfusion.

This sub-study is also notable for the careful, systematic process by which preoperative and postoperative CTA data were analysed by an independent core imaging laboratory. One prior study has reported similar radiographic results among 16 patients with preoperative cerebral malperfusion treated with AMDS at a single centre [17]. However, the prior report was derived from a single-centre cohort using local imaging reports, so we posit that the independent, systematic assessment of imaging data in our study represents a strength that also augments confidence in the findings of the prior report. Despite the promising results of the study and our ability to report quantitative data regarding these imaging findings, there remains room for improvement in future studies. For example, a number of postoperative CTAs were of poor quality based on contrast timing or were not obtained at the intended time points. While this to some extent reflects ‘real-world’ management of these complex patients, future trials should focus efforts on standardizing imaging protocols and optimizing subject follow-up. We hope the PERSEVERE trial can set a new standard in regards to the objective study of imaging in aortic disease upon which future trials can improve.

The European Association for Cardio-Thoracic Surgery (EACTS), in conjunction with The STS, recently published guidelines for the diagnosis and management of aortic dissection [18]. These guidelines make clear that maneuvers that enhance the likelihood of achieving maximal TL perfusion are strongly recommended. These strategies include axillary perfusion, circulatory arrest with cerebral perfusion and evaluation (and, when indicated, treatment) of the distal arch and descending aorta for entry tears. Thus, hemiarch replacement with exclusion or resection of the primary entry tear (with or without adjuncts depending on the clinical scenario) is recommended as the standard of care by these guidelines in all patients with type A dissection. The role of elephant trunk or frozen elephant truck extension remains to be defined, but the data regarding the AMDS prosthesis reported in this study and others [8, 19] suggest this device warrants further consideration and evaluation in patients presenting with ADTI particularly in cases where preoperative malperfusion is evident and the primary entry tear is the in ascending.

This study is subject to several limitations, some of which we have already mentioned. The small sample size of only 30 patients represents a key limitation. However, it is important to note that preoperative cerebral malperfusion is a relatively uncommon diagnosis in patients with type A aortic dissection—even the most recent report available from the comprehensive IRAD database regarding preoperative cerebral malperfusion included only 362 patients [4]. The lack of a control group enrolled in the prospective trial limits our ability to compare outcomes with the study device against similar patients, but robust reference cohorts were available for comparison. Nevertheless, we report prospectively collected data with imaging data that was uniquely and objectively assessed by an independent core laboratory. These factors represent strengths compared to the majority of literature regarding management of ADTI, which is typically retrospective in nature. This sub-study also reports a relatively short follow-up period, but we argue this period adequately captures the timeframe in which patients with ADTI are most likely to suffer neurologic complications. Nevertheless, long follow-up is necessary to understand the implications of ADTI repair with the AMDS device and whether continued positive remodelling of the aortic branch vessels occurs beyond the current study period.

Finally, there were limitations regarding the quality and timing of pre- and postoperative imaging. Although 2 patients were graded to have newly patent FL channels in their postoperative imaging, it is possible that preoperative cross-sectional imaging was obtained with poor timing or a lack of a delayed phase and in the setting of low cardiac output. Suboptimal imaging protocols in this setting may have resulted in these patients’ preoperative scans being graded with partially thrombosed FL when in reality these channels were patent. Additionally, the quality of preoperative imaging precluded definitive analysis of whether malperfusion was static or dynamic in nature. Because preoperative cross-sectional imaging of the head and neck was not required for trial enrollment (and was not commonly obtained in the routine clinical work-flow of patient suspected to have aortic dissection), we were unable to analyse for radiographic changes in the right common carotid artery.

## CONCLUSION

Patients presenting with ADTI and cerebral malperfusion represent a uniquely challenging pathology. Ascending/hemiarch replacement remains the standard of care. Based on the results of this, unspecified exploratory interim analysis of the PERSEVERE trial, the AMDS device may serve as an adjunct to reduce postoperative neurologic complications and to promote positive remodelling of the IA and LCCA in patients with ADTI and cerebral malperfusion. Additional studies will be necessary to confirm these hypothesis-generating findings.

## Data Availability

Dataset is from an ongoing investigational clinical study, which is continuing to collect further follow-up data; data are not provided.

## ABBREVIATIONS

ADTI: Acute DeBakey type I dissection
CTA: Computed tomography angiography
DANE: Distal anastomotic new entry tears
FDA: Food and Drug Administration
FL: False lumen
IA: Innominate artery
IDE: Investigational Device Exemption
IRAD: International Registry of Aortic Dissection
MAE: Major adverse event
LCCA: Left common carotid artery
STS: Society of Thoracic Surgeons
TL: TAD: True lumen to total arterial diameter ratio
